# The Vaginal Microbiome Changes During Various Fertility Treatments

**DOI:** 10.1007/s43032-024-01484-0

**Published:** 2024-02-20

**Authors:** M.M. van den Tweel, E.H.A. van den Munckhof, M. van der Zanden, A. Molijn, J.M.M. van Lith, K.E. Boers

**Affiliations:** 1 Department of Obstetrics and Gynaecology, Leiden University Medical Center, Leiden, The Netherlands; 2 Department of Obstetrics and Gynaecology, Haaglanden Medical Center, Bronovolaan 5, 2597 AX The Hague, The Netherlands; 3 DDL Diagnostic Laboratory, Rijswijk, The Netherlands; 4 Eurofins NMDL-LCPL, Rijswijk, The Netherlands

**Keywords:** Bacterial vaginosis, Microbiome, Hormone treatment, IVF, IUI

## Abstract

This study aimed to investigate the influence of hormonal treatment on the vaginal microbiome during fertility treatments. Bacterial vaginosis (BV) could affect fecundity, particularly in the in vitro fertilization (IVF) population, where negative effects on pregnancy outcomes have been reported. It is hypothesized that the hormone treatment during fertility treatments could influence the abundance of *Lactobacilli*, with negative effects on the pregnancy results. A total of 53 couples attending a fertility clinic in the Netherlands between July 2019 and August 2022 were included in this prospective cohort study. Vaginal samples were collected at start of treatment, oocyte retrieval or insemination from subjects undergoing intra uterine insemination (IUI) with mild ovarian stimulation, and IVF or intra cytoplasmatic sperm injection (ICSI) with controlled ovarian hyperstimulation. AmpliSens® Florocenosis/Bacterial vaginosis-FRT qPCR and 16S rRNA gene-based amplicon sequencing were performed on all samples. In total, 140 swabs were analyzed, with a median of two swabs per person. 33 (24%) tested qPCR BV positive. *Lactobacilli* percentage decreased during fertility treatments, leading to changes in the vaginal microbiome. Shannon diversity index was not significantly different. Of the total of 53 persons, nine switched from qPCR BV negative to positive during treatment. The persons switching to qPCR BV positive had already a (not significant) higher Shannon diversity index at start of treatment. If the vaginal microbiome of persons deteriorates during fertility treatments, timing of following treatments, lifestyle modifications, or a freeze all strategy could be of possible benefit.

**Supplementary Information:**

The online version contains supplementary material available at 10.1007/s43032-024-01484-0.

## Introduction

The vaginal microbiome is known to change over time, depending on menstrual cycle, suggesting that the vaginal microbiome is influenced by hormones. *Lactobacilli* are bacteria known to be dependent on hormonal status, especially levels of estrogen, in women. Lack of *Lactobacilli* is associated with an abnormal microbiome, also called bacterial vaginosis. Incidence of bacterial vaginosis (BV) can be as high as 29%, with a significant portion of persons being asymptomatic [1, 2].

It is hypothesized that the hormones used during assisted reproduction could influence the vaginal microbiome through glycogen accumulation, which is utilized by *Lactobacillus* [2, 3]. Recent studies in persons undergoing in vitro fertilization (IVF) have shown a negative effect of an abnormal microbiome on pregnancy outcomes [4, 5]. Over the past decade, more research has been published on the endometrial and vaginal microbiome, with a growing understanding of a continuum between the vaginal and endometrial microbiome [6].

Only a few studies have investigated the influence of external hormones on the microbiome over time. One study found no effect of vaginal progesterone treatment during pregnancy on vaginal microbiome [2] and another study reported no microbiome changes during IVF treatment [7]. Other studies reported alterations in the vaginal microbiome during IVF; however, these studies were small [3, 8]. Carosso reported a small decrease in *Lactobacilli* and higher bacterial diversity during the first IVF treatment session compared to pretreatment. Hyman found changes in microbiome status during IVF; however, all subjects were treated with antibiotics, which could also influence their microbiome. There is currently no literature on the impact of lower dose hormonal stimulation during intra uterine insemination (IUI) cycles on the vaginal microbiome.

This is the first study monitoring the vaginal microbiome using qPCR and sequencing during multiple consecutive fertility treatments (pretreatment and around ovulation) in a subfertile population undergoing IUI, IVF, and intracytoplasmic sperm injection (ICSI). It is hypothesized that *Lactobacilli* will decrease over time, potentially leading to a higher incidence of bacterial vaginosis.

## Materials and Methods

This study was performed on a prospective single center cohort in the Haaglanden Medical Center (HMC) in the Hague, the Netherlands. The fertility department of the HMC collaborates with the IVF laboratory of the Leiden University Medical Center. Persons (18 years or older) undergoing fertility treatments directly after fertility assessment were included between July 2019 and June 2022. Exclusion criteria were inability to understand Dutch or English language, three or more miscarriages, and prophylactic antibiotic treatment. No couples were treated with donor sperm or donor oocytes. Microbiological assessment of vaginal swabs was done by external laboratory of Eurofins NMDL and DDL Diagnostic Laboratory in Rijswijk, The Netherlands.

### Sample and Data Collection

When persons signed informed consent at their initial fertility assessment (intake), the first vaginal swab (e-swab, Copan Italia SpA, Breschia, Italy) was taken from the posterior fornix after inserting a speculum. No measurements were performed if the participants were menstruating, on hormonal therapy or post-coital. At consecutive IUI or IVF/ICSI procedures the sampling was performed prior to the ovum pick up or insemination, up to a maximum of four procedures. The vaginal sampling was performed at moment of the last ultrasound before transfer in case of a frozen embryo cycle. No new swabs were collected after an ongoing pregnancy or when switched from IUI to IVF/ICSI. The swabs were used for BV qPCR and microbiota analysis. Fertility doctors and study participants were blinded for the results of the swab. Participants with symptoms of BV were tested separately and treated according to the standard protocol when tested positive.

Follow-up ended at cessation of treatment, ongoing pregnancy or at end of study (August 2022). Data about patient characteristics and fertility treatments were extracted from the electronic patient files. A cloud-based clinical data management service, Castor EDC, was used to collect all data.

### Fertility Treatments

All couples starting with IUI or IVF/ICSI at the HMC hospital need to stop smoking before starting treatment and need to have a body mass index (BMI) below 35. Fertility treatment is initiated if indicated based on the results of the fertility assessment. Couples with unexplained infertility started with IUI with mild ovarian stimulation or IVF based on female age. For IVF/ICSI protocols, both long and short agonist and antagonist protocols were applied. Estrogen (E2) levels are measured in blood in most cases before ovum pick up is scheduled. During ovum pick up, antibiotics are not routinely administered, but only given for certain indications (for example with endometriomas).

### Nucleic Acid Extraction

In the HMC laboratory, the vaginal samples were frozen within 24 h and afterwards transported to the external laboratory for molecular analysis. DNA was extracted from 200 μl samples and eluted in a final volume of 100 μl with the MagNA Pure 96 instrument using the MagNA Pure 96 DNA and Pathogen Universal small Volume Kit and the Pathogen Universal protocol (Roche Diagnostics, Basel, Switzerland). Appropriate positive and negative control samples were included in testing, which were evaluated in the BV qPCR.

### BV qPCR

The extracted DNA was tested according to the manufacturer’s instructions with a CE-IVD marked multiplex quantitative PCR assay (the AmpliSens® Florocenosis/Bacterial vaginosis-FRT PCR kit InterLabService, Moscow, Russia). Based on the presence of *Lactobacillus* species, *Gardnerella vaginalis*, *Atopobium vaginae* (recently reclassified as *Fannyhessea vaginae*) [9], and total amount of bacteria, swabs were labeled as BV positive (amount of *G. vaginalis* and/or *A. vaginae* is almost equal or exceeds the amount of *Lactobacillus*), BV negative (*G. vaginalis* and/or *A. vaginae* are absent or its amount is considerable less than the *Lactobacillus* amount), unspecified dysbiosis (amount of *Lactobacillus* is reduced relative to the total amount of bacteria, whereas *G. vaginalis* and/or *A. vaginae* are absent or its amount is considerable less than total amount of bacteria), or suspected dysbiosis (amount of *G. vaginalis* and/or *A. vaginae* is similar to the amount of *Lactobacillus* but does not exceed the limit value) using the software tool provided by the kit manufacturer.

### Microbiota Analysis

Microbiota analysis was performed on the extracted DNA of swabs of persons with repeated measurements of vaginal microbiome. A fragment of ~421bp of the V1–V2 region of the 16S rRNA gene was amplified using the primers described by Ravel et al. and Walker et al. with Illumina overhang adaptor sequences added [10, 11]. Outcomes were categorized in one of five vaginal microbiome community state types (CST), as reported by Ravel et al. CST I is dominated by *L. crispatus*, and respectively CST II by *L. gasseri*, CST III by *L. iners*, CST IV by non-lactobacilli, CST V by *L. jensenii* [11]. Previously described literature on this subject also used these CSTs. More details on the microbiome analysis are described in the attachment.

### Outcomes

Primary outcome were *Lactobacilli* amount and Shannon diversity index. Secondary outcomes were change of qPCR BV status or microbiome CST. Shannon diversity index (SDI) is a commonly used weighted measure to analyze the various types of bacteria in a particular environment. The SDI is zero when there is only one type of bacteria present and there is no diversity [12]. Sample size was not calculated upfront. For statistical analysis, IBM SPSS statistics for Macintosh, version 27, was used. Main outcome parameters were analyzed using a paired *t* test.

## Results

### Population

**Fig. 1:**
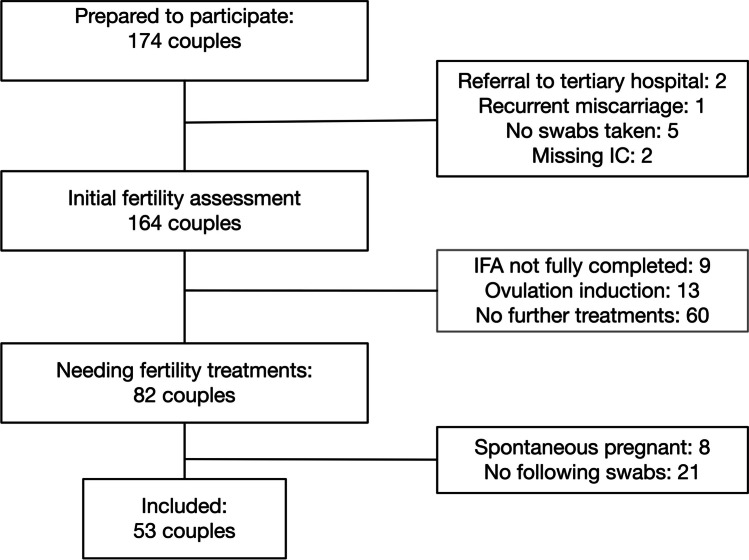
Flow diagram of included participants

In total, 82 couples were eligible for inclusion; however, of 21 participants, there were no following swabs, and eight participants became pregnant spontaneously before starting fertility treatment. In Fig. 1, the included participants are shown. The remaining couples were multi-ethnic and most had a high socioeconomic status (Table 1). Only two persons had discharge complaints. Twenty-two persons were treated with IVF and 31 persons treated with IUI. In total 53 participants had 87 consecutive treatment cycles (in total 140 samples), with a median of two samples per person.

**Table 1:** Baseline characteristics of participants at intake

Participants (descriptives at time of initial fertility assessment)	53
Age (mean, SD)	34.9 (4.3)
Discharge complaints *n*(%)	2 (4%)
Antibiotic/antifungal treatment *n*(%)	1 (2%)
BMI (mean, SD)	25 (4.3)
Smoking *n*(%)	5 (9%)
Alcohol (≥ 1 glass a week) *n*(%)	16 (30%)
Drug use (on regular basis) *n*(%)	3 (6%)
HPV positive in past year *n*(%) (only tested when indicated)	9 (17%
Medication use (for comorbidities)	6 (11%)
Ethnicity *n*(%)*	
Caucasian	30 (57%)
Hindu	4 (7.3%)
African	1 (2%)
Antillean	4 (7.3%)
Asian	6 (11%)
Moroccan	3 (6%)
Turkish	1 (2%)
Other	4 (7.3%)
Socioeconomic status *n*(%)**	
Low	0
Middle	15 (28%)
High	37 (70%)
Missing	1 (2%)
Regular cycle *n*(%)	49 (92%)
Subfertility duration in years (median, IQR)	1 (1–2)
Cause of subfertility *n*(%)***	
Male factor	19 (36%)
Tubal factor	6 (11%)
Hormonal	3 (6%)
Endometriosis	7 (13%)
Unexplained	16 (30%)
Other	2 (4%)
Fertility treatment	
Intra uterine insemination (IUI)	31 (58%)
In vitro fertilization (IVF)	22 (42%)

*Other mostly Hispanic participants

**As defined by education status

***Hormonal: premature ovarian insufficiency or anovulation, other: uterine myoma’s, uterus anomaly, sexual disfunction

### Microbiota Over Time

Of the 140 samples, 33 (24%) were tested positive for BV qPCR. At intake, 10 of 53 (19%) persons tested BV qPCR positive; and during treatment, 17 of the 53 (32%) tested BV qPCR positive. The 140 samples were also tested with the microbiota analysis (Supplemental figure 1). The details about the microbiome results and the comparison with qPCR are described in another paper [13].

**Fig. 2:**
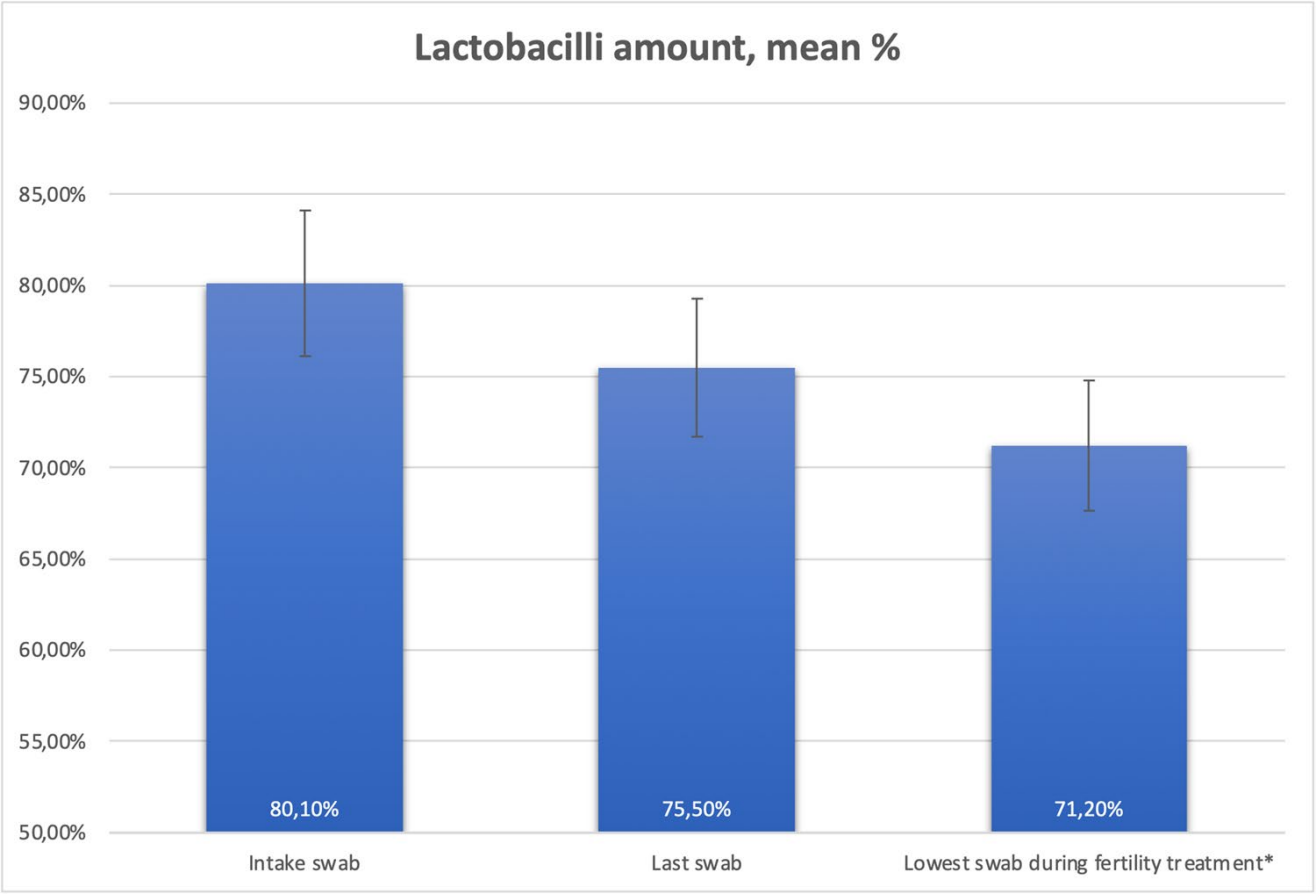
Decrease of *Lactobacilli* during fertility treatment (*p = 0.01)

On average, the number of *Lactobacilli* decreased 4.6% from intake to last treatment, and 8.9% when comparing the intake swab with the lowest following swab during treatment. This decrease in *Lactobacilli* was significant (*p* = 0.01, Fig. 2). Shannon diversity index (SDI) was not significantly different from first sample (*p* = 0.59). During fertility treatment, more than 10% decrease of *Lactobacilli* can be seen in 17 of the 53 persons (and only a 10% increase in four persons). This appeared in all community state types (CST). E2 levels were measured in most IVF participants but did not show a specific association with changing microbiome (data not shown).

**Fig. 3:**
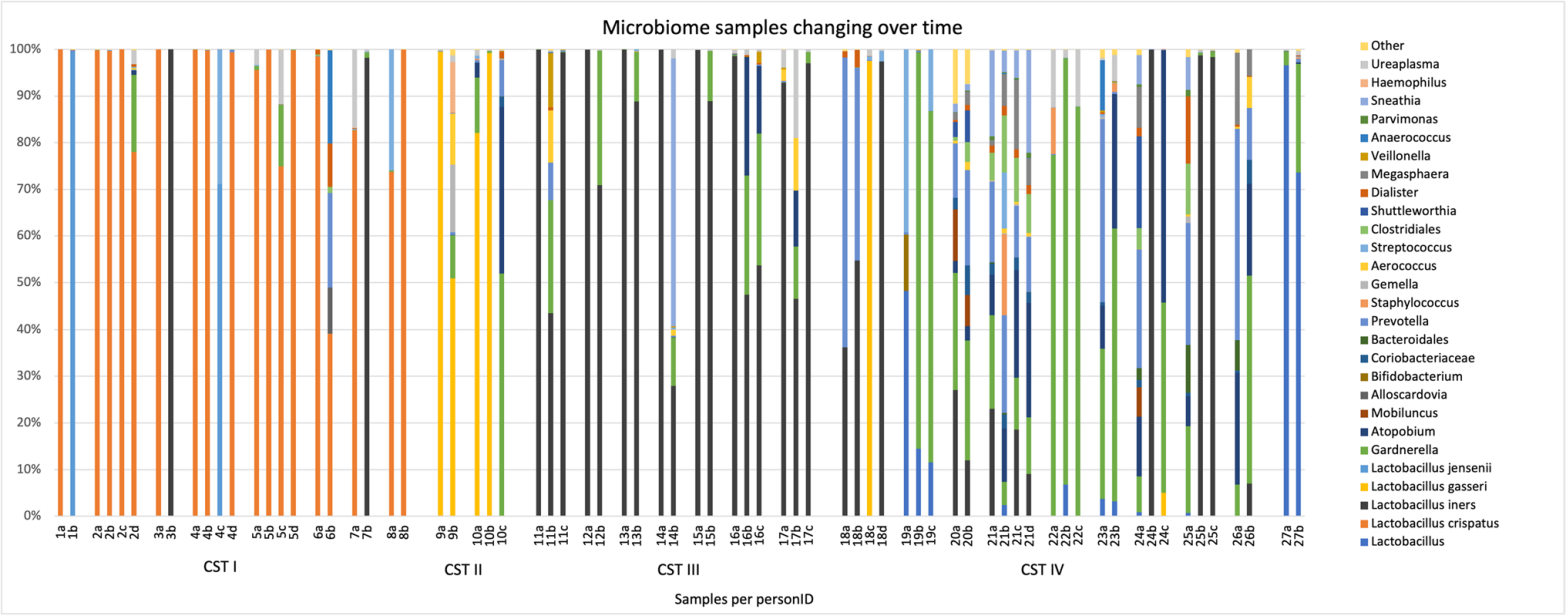
The microbiome of 27 persons with a more than 10% change in *Lactobacilli*, *Gardnerella*, or *Atopobium/Fannyhessiae* over time. Samples are arranged per person, categorized on community state type microbiome on basis of the first sample at intake

Of the 52 participants, in total, 27 persons had a more than 10% change in *Lactobacilli*, *Gardnerella*, or *Atopobium/Fannyhessiae* over time (Fig. 3). Of these 27 persons, eight, two, seven, and nine persons were classified as CST I, II, III, and IV at intake, respectively. One participant had a microbiome dominated by *L. ultenesis/L. johnsonii* and could not be classified in the regular CST (in Fig. 3 personID 27).

### Deterioration of the Microbiota

In total, 13 persons had a deterioration of qPCR or CST result (including switching to less favorable *L. iners*) as shown in Table 2 (extended descriptions of which bacteria leaded to a change is shown in Supplement Table 1). Nine of these persons had IVF/ICSI treatment and four had IUI treatment. The decrease of *lactobacilli* resulted in a switch from BV negative to BV positive in nine persons (personID 10 at second treatment). There were no differences seen compared to the whole group based on ethnicity, reason of subfertility, gonadotropin dose, or IVF protocol (not all data shown). However, five of 14 negatively changed persons had human papillomavirus (HPV) infection last year. Also, two persons were (former) smokers. In swabs that switched from qPCR BV negative to positive, there were already a small number of non-lactobacilli bacteria present in the vaginal microbiome at baseline. The mean Shannon diversity index was 1.02 in the qPCR BV negative baseline samples who switched and 0.87 in the baseline samples that remained qPCR negative (not significant *p* = 0.46). The main non-lactobacilli bacteria found in these swabs were *Gardnerella* or *Atopobium/Fannyhessiae*.

**Table 2:** Detailed description of persons with changed microbiome

PersonID	qPCR at intake	CST at intake	Day of menstrual cycle	SDI at intake	Treatment	2nd qPCR	2nd CST	Day of menstrual cycle2	Gonadotropin dose (IU)	E2 level (pmol/L)	3rd qPCR	3rd CST	Day of menstrual cycle22	Gonadotropin dose2	E2 level2	Related factors
1	neg	I	28	1.69	IVF	neg	V	11	300	3632						HPV +
*Deteriorated microbiota*																
3	neg	I	14	0.63	IUI	neg	III	15	37.5							
5	neg	I	9	1.55	IUI	pos	I	12	62.5		neg	I	12	62.5		antibiotics for UTI, HPV +
6	neg	I	15	1.25	IVF	neg	IV	22	112.5	7073						stopped smoking, HPV +
7	neg	I	13	2.07	ICSI	neg	III	14	225	8201						stopped smoking, HPV +
9	neg	II	8	1.39	IVF	pos	II	18	225	4848						
10	pos	II	2	2.55	IUI	neg	II	13	50		pos	IV	12	62.5		
11	neg	III	8	0.23	ICSI	pos	IV	15	300	6133	neg	III		300	6154	HPV +
12	neg	III	14	0.78	ICSI	pos	III	14	150	7650						
14	neg	III	21	0.6	IVF	neg	IV	17	150	4149						
15	neg	III	16	0.12	ICSI	pos	III	27	112.5							HPV +
16	neg	III	8	0.14	IUI	pos	IV	13	50		pos	III	11	75		
17	neg	III	4	0.52	IVF	pos	IV	13	200	2879	neg	III	13	n.a., frozen embryo transfer		
19	neg	IV	6	1.46	IVF	pos	IV	12	225	4521	pos	IV	13	225		
*Improved microbiota*																
18	neg	IV	13	2.43	IUI	neg	III	11	75		neg	II	9	87.5		antibiotics for discharge
24	pos	IV	13	4.15	IVF	neg	III	22	150	1866	pos	IV		urinary FSH 225	3711	decreased BMI
25	pos	IV	15	3.85	IUI	neg	III	12	62.5		neg	III	8	62.5		stopped smoking

In one person (personID 12), *Staphylococcus* and *Ureaplasma* were found and *Ureaplasma* and *Gardnerella* increased during treatment. In another person (personID 19), mainly *Bifidobacterium*, *Streptococcus*, and *Prevotella* were found, and *Gardnerella* was increased during treatment. One subject changed to qPCR BV positive during one of the IUI cycles (personID 5) just after an antibiotic treatment for a urinary tract infection. One person (personID 17) had a dramatic decrease of *Lactobacilli* during IVF treatment and switched to qPCR BV positive. This person turned back to qPCR BV negative during her frozen embryo transfer in her natural cycle. In total, 11 persons switched from microbiome CST. Two persons (personID 3 and 7) who changed from CST I to CST III had already a low amount of *L. iners* in the first swab, one of them stopped smoking (personID 7).

### Improvement of the Microbiota

Three persons had an improvement of their microbiome. Two persons switched from qPCR BV positive to negative during IUI treatments. One (personID 18) switched to qPCR BV negative because of treatment with antibiotics for discharge complaints. The other person (personID 25) stopped smoking before starting treatment. One person switched (personID 24) from qPCR BV positive to negative during first IVF treatment. The person lost some weight prior to start with IVF (BMI below 35 at start IVF). With the second IVF stimulation, this person switched back to qPCR BV positive again. A different stimulation protocol with urinary FSH was used this second time, compared to recombinant FSH the first stimulation.

## Discussion

This is the first study investigating the impact of hormones during multiple fertility treatments on the vaginal microbiome. This study showed that fertility treatment resulted in a decrease in the rate of *Lactobacilli,* ranging from 4.6 to 8.9% in the worst case. Shannon diversity index did not show a significant difference, which suggests that the decrease in *Lactobacilli* is most prominent in samples that were already more diverse at intake. At intake, 10 of 53 (19%) persons tested BV qPCR positive; and during treatment, 17 of the 53 (32%) tested BV qPCR positive. In total, 13 persons showed a deterioration in their BV PCR and/or microbiome result (including changing to less favorable *L. iners*). In contrast, only three persons showed an improvement of their microbiome status. This could be probably intentional because these persons were trying to improve their lifestyle.

A strength of this study are the multiple observations during consecutive treatment cycles. Earlier studies only examined the microbiome prior to IVF treatment and during the first treatment, with less than 30 participants [3, 7, 8]. Carosso et al. did find a decrease of *Lactobacilli*; however, this was not statistically significant. Possibly, *Lactobacilli* decrease more pronounced after undergoing multiple treatments. Another strength is the heterogenous population studied, which includes non-Caucasian persons and a mix of IUI and IVF/ICSI treatment protocols. This enhances the applicability of the findings and allows for a broader understanding of the potential impact of fertility treatments on the vaginal microbiome.

This decrease in *Lactobacilli* and negative change of microbiome status could have a negative effect on ongoing pregnancy rates. Another paper by this research group showed that samples with a consecutive miscarriage had 15% less *Lactobacilli* compared to samples with a consecutive ongoing pregnancy (however not significant) [13]. The numbers in this study were too low to calculate specific pregnancy outcomes for changing qPCR or microbiome status compared to persons with a stable microbiome. It should be further investigated which amount of decrease of *Lactobacilli* is clinically relevant. Understanding the fluctuations of the vaginal microbiome and knowing which persons are at risk could improve fertility treatments in the future.

This study is the largest study conducted on this subject to date; however, the numbers of subjects is still limited because this study is a sub-analysis of a larger prospective study. This study did not consider sexual intercourse and the seminal microbiome around time of sampling, which could influence the change of vaginal microbiome as well. Another limitation could be the variability in hormonal treatment protocols utilized. The most common stimulation was with recombinant gonadotropins, and an agonist protocol in IVF*.* However, the decrease of *Lactobacilli* was observed in both IUI and IVF, suggesting that it is not solely depending on specific protocols (recombinant or urinary FSH, antagonist or agonist protocol) or on E2 levels, as previously suggested by Hyman et al.

The change of vaginal microbiome could be related to overall health status or the immune system. The persons with a deteriorated microbiome during treatment were found to be more frequently smokers and/or tested positive for HPV (human papillomavirus). The correlation between HPV infections and abnormal microbiota has already been reported in literature before [14]. Healthier lifestyle might have a positive impact on the microbiome. Three cases presented here show this positive effect; however, it is important to note that these examples are anecdotal, and this is insufficient evidence for a broader conclusion. If lifestyle changes could reduce recurrence of BV after treatment or prevent the worsening of the microbiome during fertility treatment, this could serve as additional motivation for persons to adopt healthier lifestyle before conception.

Vaginal microbiome testing to monitor changes in *Lactobacilli* levels during IUI or IVF treatment could provide valuable insights. Future research should focus on the microbiome and pregnancy results in the context of multiple sequential fertility treatments. This study showed one case in which the vaginal microbiome returned to normal during frozen embryo transfer (personID 17). If future research can show a clinically relevant association between the vaginal microbiome and pregnancy outcomes, it may lead to different treatment strategies. Treatment options such as freeze all strategy, introducing pauses between treatments, vaginal probiotics, and lifestyle interventions could be investigated further to improve pregnancy outcomes.

### Supplementary Information

Supplementary file 1 Supplemental Fig. 1: all microbiome samples arranged per person, categorized on community state type microbiome on basis of their first sample at intake. Persons used in Figure 3 are marked with their personID in this figure as well. (TIFF 62458 kb)

Supplementary file 2 Supplemental table 1: detailed description of persons with changed microbiome (XLSX 10 kb)

Supplementary file 3 (DOCX 27 kb)

## Data Availability

The data underlying this article will be shared on reasonable request to the corresponding author.
