# Supplementary figures and images for: The Vaginal Microbiome Changes During Various Fertility Treatments
Source: Reprod Sci. 2024 Feb 20;31(6):1593–600. doi: 10.1007/s43032-024-01484-0 (PMC11111482; doi:10.1007/s43032-024-01484-0)

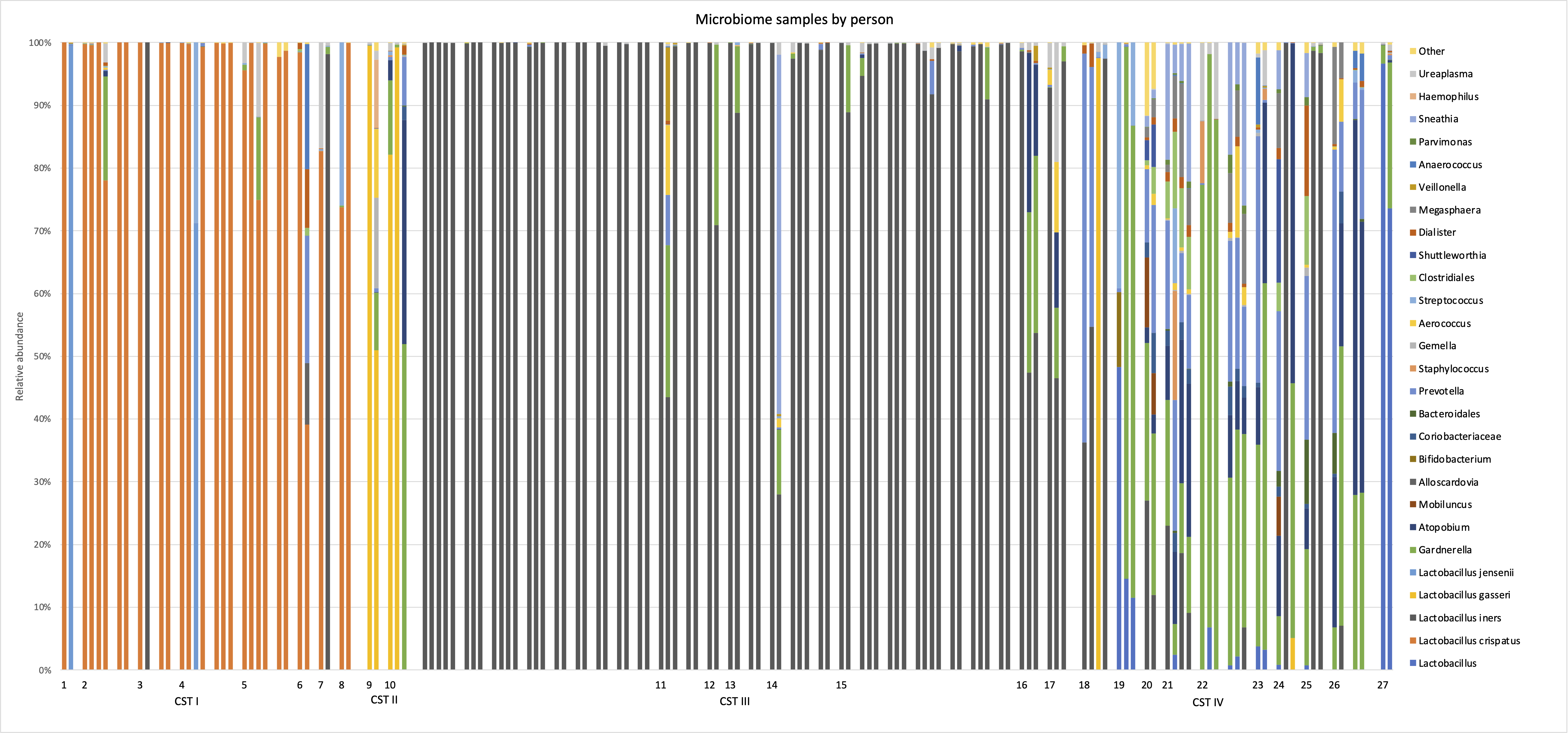

Supplement: Supplementary file 1 — Supplemental Fig. 1: all microbiome samples arranged per person, categorized on community state type microbiome on basis of their first sample at intake. Persons used in Figure 3 are marked with their personID in this figure as well. (TIFF 62458 kb) [file 43032_2024_1484_MOESM1_ESM.tiff]
