# Supplementary material for: The Vaginal Microbiome Changes During Various Fertility Treatments
Source: Reprod Sci. 2024 Feb 20;31(6):1593–600. doi: 10.1007/s43032-024-01484-0 (PMC11111482; doi:10.1007/s43032-024-01484-0)
Supplement: Supplementary file 3 — (DOCX 27 kb) [file 43032_2024_1484_MOESM3_ESM.docx]

# The vaginal microbiome changes during various fertility treatments

**Drs. M.M. van den Tweel^ab^, dr. E.H.A. van den Munckhof^c^, dr. M. van der Zanden^b^, drs. A. Molijn^d^, prof. J.M.M. van Lith^a^, dr. K.E. Boers^b^**

aDepartment of Obstetrics and Gynaecology, Leiden University Medical Center, Leiden, The Netherlands; bDepartment of Obstetrics and Gynaecology, Haaglanden Medical Center, The Hague, The Netherlands; ^c^ DDL Diagnostic Laboratory, Rijswijk, The Netherlands; ^d^ Eurofins NMDL-LCPL, Rijswijk, The Netherlands

**Corresponding author:** Dr. K.E. Boers, kim.boers@haaglandenmc.nl, Bronovolaan 5, 2597 AX The Hague, The Netherlands. Phone number secretary K.E. Boers: + 31 88 979 4496.

# Supplement

Each 50 µL PCR reaction contained 5 µL (10x) Expand High Fidelity Buffer with 15 mM MgCl2 (Roche), 2.6 U Expand High Fidelity Enzyme mix (Roche), 0.2 mM of each dNTP (Roche), various primer concentrations and 10 µL of extracted DNA. The PCR was run for 2 min at 94°C followed by 35 cycles of 94°C for 15 sec, 55 °C for 30 sec and 72 °C for 1 min and a final extension step at 72 °C for 7 min. The PCR products with a visible band of ~421bp on gel were subsequently purified and quantified using AMPure XP Beads (Agencourt Bioscience Corporation, Beverly, USA) and the Quant-iT PicoGreen dsDNA Assay Kit (Invitrogen, Paisley, UK), respectively. After library preparation using the Nextera XT kits (Illumina, San Diego, USA), sequencing was performed with the MiSeq desktop sequencer using the MiSeq Reagent Kits v2 500-cycles (Illumina). In each PCR run, a microbial community standard (ZymoBIOMICS) was included as positive control and PBS as negative control. Both controls were assessed on gel and included in the sequencing run to assess the quality. Samples should have a minimum number of 80,000 reads per sample and at least 75% of the reads should have an average quality score (Phred) ≥ Q30 to continue with data analysis. The QIIME pipeline was used to process sequencing data. [13]Open reference operational taxonomic units clustering of high-quality sequences (≥ 100bp in length with a quality score ≥ Q20) was conducted at a 97% similarity level against a pre-clustered version of the Augustus 2013 GreenGenes database. No low abundance filtering was used. Instead OTUs were checked for relevance per sample. Low abundance OTUs that were not relevant for any sample were included in the group “others”.

Determining the CST-classification

The highest percentage of type of Lactobacillus was chosen to determine to which CST the sample belonged. For example, if a sample contained 60% of *L. iners* and 30% of *L. jensenii*, the sample was classified to CST III (*L. iners*). Samples having less than 50% *Lactobacilli* were classified as CST IV.
